# Health Beneficial Bioactivities of Faba Bean Gastrointestinal (In Vitro) Digestate in Comparison to Soybean and Pea

**DOI:** 10.3390/ijms23169210

**Published:** 2022-08-16

**Authors:** Delphine Martineau-Côté, Allaoua Achouri, Janitha Wanasundara, Salwa Karboune, Lamia L’Hocine

**Affiliations:** 1 Agriculture and Agri-Food Canada, Saint-Hyacinthe Research and Development Centre, Saint-Hyacinthe, QC J2S 8E3, Canada; 2 Department of Food Science and Agricultural Chemistry, Macdonald Campus, McGill University, Sainte-Anne-de-Bellevue, QC H9X 3V9, Canada; 3 Agriculture and Agri-Food Canada, Saskatoon Research and Development Centre, Saskatoon, SK S7N 0X2, Canada

**Keywords:** faba bean, pulse protein, bioactive peptides, gastrointestinal digestate, antioxidant, antidiabetic, Dipeptidyl Peptidase-IV inhibitors, antihypertensive, Angiotensin-Converting Enzyme inhibitors

## Abstract

Faba beans are a promising emerging plant-based protein source to be used as a quality alternative to peas and soy. In this study, the potential health beneficial activities of three Canadian faba bean varieties (Fabelle, Malik and Snowbird) were investigated after in vitro gastrointestinal digestion and compared to two commonly used legumes (peas and soy). The results revealed that the faba beans had a higher antioxidant activity than peas when assessed with the 2,2-diphenyl-1-picrylhydrazyl (DPPH) and the 2,2′-azino-bis(3-ethylbenzothiazoline-6-sulfonic acid (ABTS) assays, except for the Fabelle variety. In the oxygen radical absorbance capacity (ORAC) and the iron chelating assays, the faba beans had a lower antioxidant activity than soy. Interestingly, Fabelle and Snowbird showed a higher antioxidant effect than the peas and soy at the cellular level. The antihypertensive properties of Fabelle and Malik varieties were significantly higher than peas but lower than soy. The in vitro antidiabetic activity was higher for soy, but no differences were found at the cellular level. The faba bean peptides were further fractionated and sequenced by mass spectrometry. Eleven peptides with in silico predicted bioactivities were successfully identified in the faba bean digestate and support validating the health-promoting properties of peptides. The results demonstrate the bioactive potential of faba beans as a health-promoting food ingredient against non-communicable diseases.

## 1. Introduction

The demand for plant-based protein sources keeps increasing to respond to environmental, ethical, health, economic and food security challenges. In these circumstances, faba beans have retained attention, due to various desirable environmental, agronomic and nutritional characteristics [1]. Indeed, faba bean production has largely increased in the last few years, from 4.5 million tons in 2014 to 5.7 million tons in 2020 [2]. Faba beans have a high protein content (~30%) [3] and are a rich source of fiber, resistant starch, minerals and vitamins [4]. Besides, novel cultivars with a low anti-nutrient content (vicine/convicine and tannins) are available [5,6], which further improves its nutritional value and application potential. 

Faba beans are also a rich source of bioactive compounds, namely dietary fibers [7,8], L-DOPA [9,10] and polyphenols [11,12], that have been associated with health benefits. The faba bean proteins could also play an important role for their health-promoting activities, by releasing small bioactive peptides after gastrointestinal digestion. The food-derived bioactive peptides possess various bio-functional capacities and can intervene in various physiological processes [13]. Their bioactive properties are beneficial in fighting against non-communicable diseases, including type II diabetes, hypertension, obesity and cancer, among others [14,15,16]. The faba bean protein hydrolysates have antioxidant [15,17,18,19,20], antidiabetic [17], antihypertensive [17], cholesterol-lowering [15,18], anticancer [15], anti-inflammatory [19] and food intake regulation properties [21] identified to date. However, the faba beans’ bioactive peptides that are released in the physiological context of gastrointestinal conditions have been scarcely investigated to date.

In this regard, the purpose of this study was to assess the health-promoting bioactive properties of three new Canadian faba bean varieties (Fabelle, Malik and Snowbird) after in vitro gastrointestinal digestion in comparison to two commonly used legumes, namely, peas and soy. The three faba bean cultivars included in the study had different quality traits: low-tannin (Snowbird); low vicine/convicine (Fabelle); and high tannin and high vicine/convicine (Malik). The raw and cooked flours were digested in vitro using a standardized gastrointestinal INFOGEST digestion protocol, to which an ileal digestion phase was added to mimic brush border digestion. The digestates were filtered on a 3 kDa molecular weight cut-off membrane to recover peptides and small bioactives. The collected filtrates were assessed for antioxidant, antidiabetic and antihypertensive activities with a combination of in vitro and cell-based assays. The bioactive properties were selected based on the results of an in silico analysis. The faba bean peptides with the highest activity were further fractionated and identified by mass spectrometry.

## 2. Results and Discussion

### 2.1. Optimization of the In Vitro Digestion Model for Subsequent Cell Culture Experiments

The impact of different in vitro digestion conditions on cell viability after incubation with the faba bean 3 kDa permeates was investigated, with the aim of determining the digestion conditions that enable acceptable cell viability, while maintaining as much as possible the standardized digestion conditions of the INFOGEST protocol. Three main digestion factors were considered: (1) the bile salts concentrations; (2) the intestinal enzymes used (pancreatin or trypsin, chymotrypsin and α-amylase, with or without the addition of a purified amino peptidase to mimic brush border digestion) and (3) the protease inhibitor concentration. A two-way ANOVA table (Table 1) was generated to explore the impact of the three factors of interest, the peptide concentrations and their interaction on cell viability at the end of the incubation period. 

**Figure 1:**
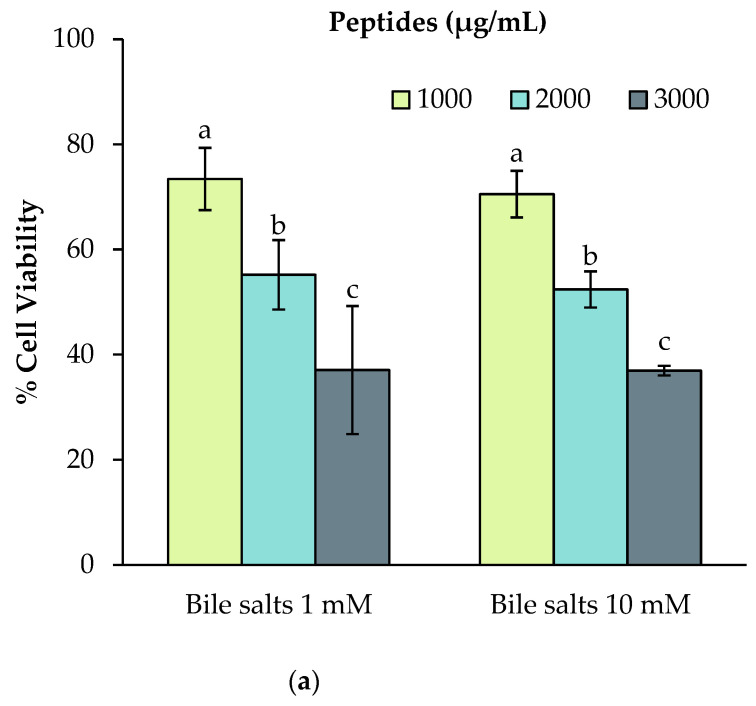

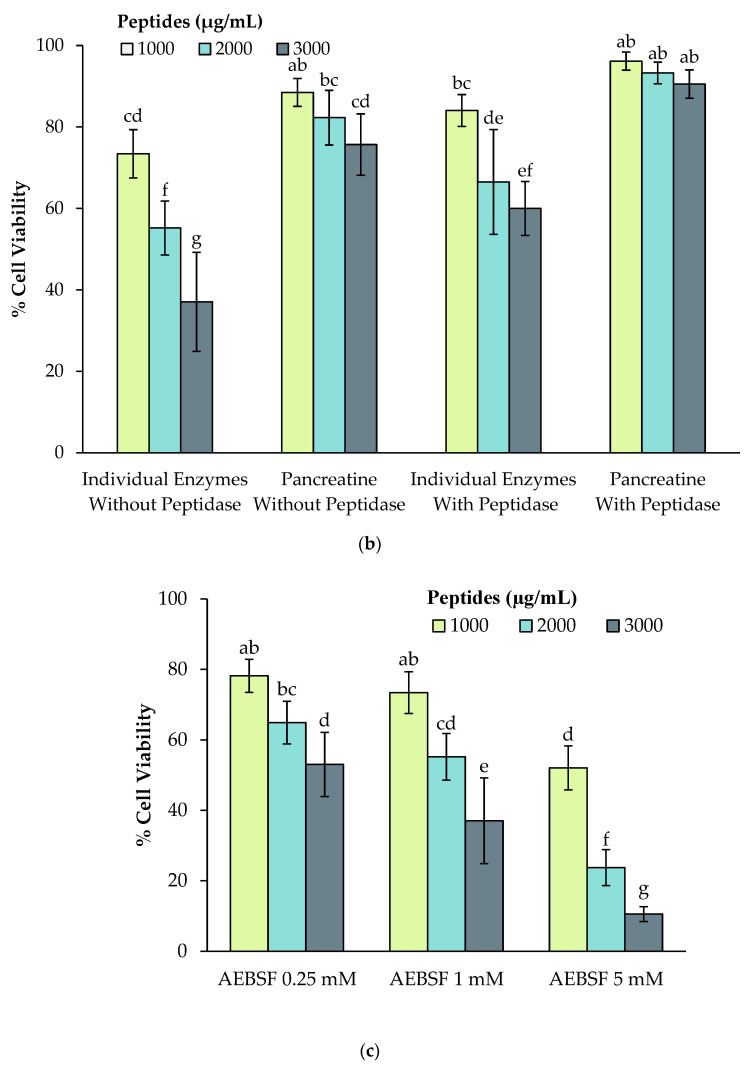
The impact of various concentrations (3000, 3500 and 4000 μg peptides/mL) of 3 kDa permeate of faba bean digestate (variety Fabelle) obtained in different digestion conditions on Caco-2 cell viability. The digestions were performed with different (**a**) bile salts concentrations; (**b**) different intestinal enzyme combinations; (**c**) different protease inhibitor (4-(2-Aminoethyl) benzenesulfonyl fluoride hydrochloride (AEBSF)) concentrations. Data are expressed as mean ± standard deviation of three experiments and means without a common letter differ (*p* < 0.05) as analyzed by two-way ANOVA and the Tukey’s test.

The results revealed that the bile salt concentration had no significant impact on the cell viability in the range of the peptide concentrations tested (Figure 1). Moreover, the use of pancreatin extract instead of individual pancreatic enzymes (namely α-amylase, trypsin and chymotrypsin) improved significantly the cell viability (*p* ˂ 0.05) (Figure 1). This result may be explained by the low purity of the porcine commercial pancreatic α-amylase used. Moreover, the pancreatic extract incorporates a broader range of pancreatic proteases (elastase, carboxypeptidase A and B), which is more physiologically relevant. The addition of the porcine intestinal peptidase to mimic brush border digestion improved the cell viability, to a value above 90% for the three peptide concentrations tested. This effect could be explained by the increase in protein digestibility, leading to a better peptide and amino acid availability for cell growth and viability.

The concentration of the protease inhibitor, 4-(2-Aminoethyl) benzenesulfonyl fluoride hydrochloride (AEBSF), used to stop the digestion also had a significant effect on cell viability (Figure 1). AEBSF reacts with the hydroxyl group of the serine side chain residue, forming a sulfonyl cross-link [22], thereby irreversibly inactivating the serine proteases [22]. Three doses of AEBSF were tested in the digestate: 0.25 mM (the recommended maximal dose for cell experiments [23]); 1 mM (the recommended dose in the first version of the INFOGEST protocol [24]) and 5 mM (the recommended dose in the INFOGEST 2.0 protocol [25]). The AEBSF dose was increased in the second version of the INFOGEST protocol version, to inactivate the residual protease activity that was found in the digestate samples [26]. The use of the 0.25 mM and 1 mM AEBSF dose had a similar impact on the cell viability (except for the 3000 ug/mL peptide dose). However, the 5 mM AEBSF dose had a significant (*p* ˂ 0.05) detrimental effect on the cell viability and is therefore not suitable for cell experiments. Since the residual protease activity was found in the digestate when the 1 mM AEBSF dose was used, we investigated whether the ultrafiltration step could remove these residual activities. Indeed, with 1 mM finale concentration of AEBSF in the digestate, 3.96 mU of residual leucine-aminopeptidase activity per mL was measured in the digestate supernatant, and no remaining activity was measured in the 3 kDa permeate. Moreover, the peptide stability in the permeate was verified during the ultrafiltration step by quantifying the free amino groups using the 2,4,6-Trinitrobenzene Sulfonic Acid (TNBS) method. No significant differences in the free amino group content were observed (*p* = 0.113). Therefore, the use of AEBSF at a final concentration of 1 mM was retained.

**Figure 2:**
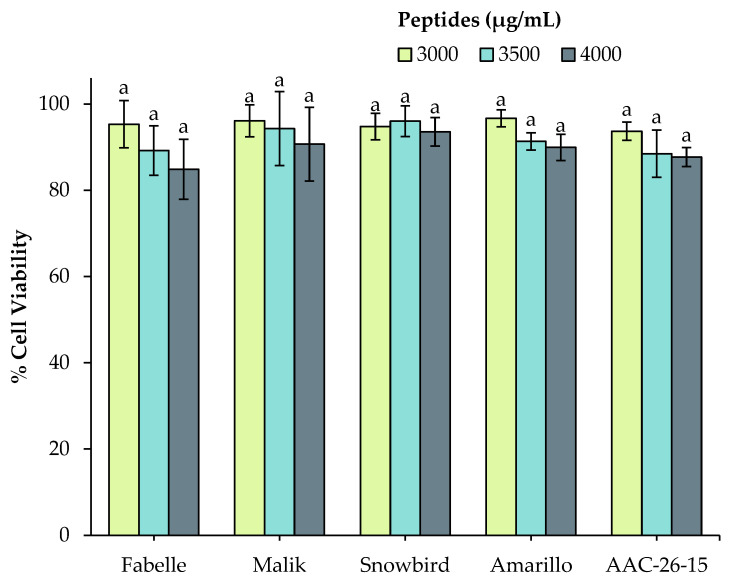
Impact of different concentrations (3000, 3500 and 4000 μg peptides/mL) of 3 kDa permeate of legume digestates obtained in the optimized digestion conditions (AEBSF 1 mM, bile salt 10 mM, pancreatin and peptidase) on cell viability. Data are expressed as mean ± standard deviation and means without a common letter differ (*p* < 0.05) as analyzed by two-way ANOVA and the Tukey’s test.

In light of these results, the use of 10 mM bile salt, pancreatin with the addition of a purified amino peptidase to mimic brush border digestion and 1 mM AEBSF during in vitro digestion were retained as well-suited conditions to enable the cell model assays, while maintaining the physiological conditions of protein digestion. The cell viability was measured after incubation with the 3 kDa permeates of each legume digestate that were obtained at these optimal digestion conditions (Figure 2). No significant differences were found in cell viability for all of the legumes at the three different tested peptide concentrations. 

### 2.2. In Silico Prediction of Bioactive Fragments Released during Gastrointestinal Digestion of Legume Proteins

**Figure 3:**
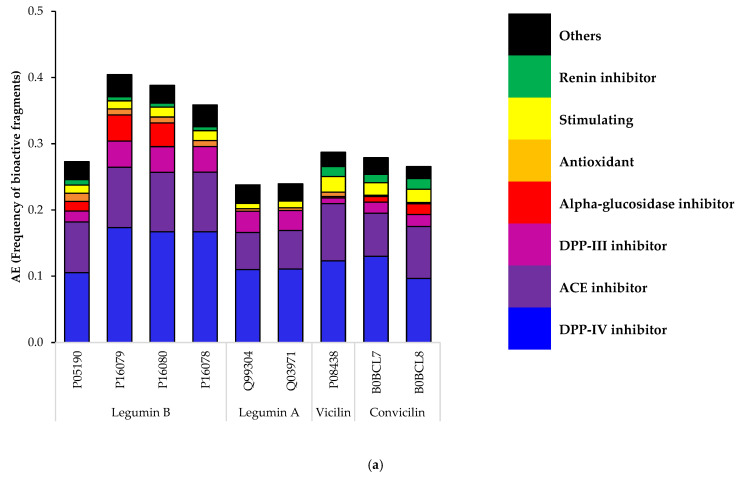

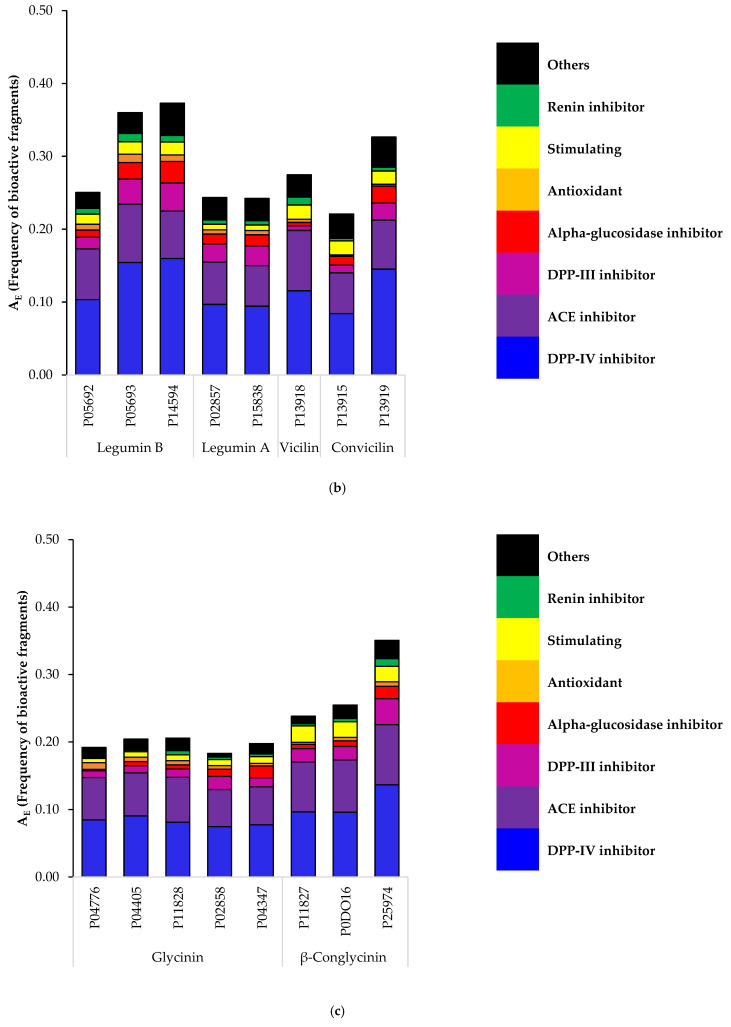
In silico prediction of bioactive fragments released during gastrointestinal digestion of (**a**) faba bean; (**b**) pea; (**c**) and soy main storage proteins.

An in silico analysis was used as a first screening tool to investigate the potential bioactive fragments released during the gastrointestinal digestion of the faba bean, pea and soy main storage proteins. As shown in Figure 3, the bioactive fragments’ frequency (A_E_) pattern was quite similar for the three legumes, which can be explained by protein sequence similarities. For faba beans and peas, Legumin B had the highest frequency of bioactive fragments, and for soy it was β-Conglycinin. The Dipeptidyl Peptidase-IV (DPP-IV) and the Angiotensin-Converting Enzyme (ACE) inhibitor were the most frequent fragments released by the selected enzymes for the three legumes. The antioxidant fragments were also present, but in a smaller proportion. It is important to note that certain peptide bioactive properties were more studied in the literature than others [27], therefore more peptide sequences with those given activities have been ascertained. When the analysis was conducted, there were 1084 ACE inhibitor, 772 antioxidant and 432 DPP-IV inhibitory fragments out of 4485 peptides listed in the Bioactive Peptide Database of University of Warmia and Mazury (BIOPEP-UWM database). The fact that less DPP-IV inhibitory fragments are listed, while they are the most frequent fragments found in the three legumes’ globulins, suggest that this is a promising bioactivity to study further. Interestingly, the frequency of the antioxidant fragments is similar to that of the more rarely studied bioactivities, such as stimulating (87), Dipeptidyl Peptidase-III (DPP-III) inhibitor (66), alpha glucosidase inhibitor (34) and renin inhibitor (41). Nonetheless, one needs to consider that the A_E_ parameter is an indication of the bioactive fragment frequency and not the bioactivity intensity. Experimental work is therefore needed to complement those results and target the most promising bioactivities. Besides, the in silico hydrolysis pattern is only based on the protein primary structure, and thus the bioactive fragments obtained experimentally are likely to differ to some extent.

Other interesting bioactivities were also present in the faba bean, pea and soy globulins, including alpha-glucosidase inhibitor, renin inhibitor and stimulating fragments. These fragments contribute to the overall antihypertensive and antidiabetic potential of the globulins. The renin is the first enzyme of the RAS system that catalyzes the conversion of angiotensinogen into angiotensin 1 [28], while the alpha-glucosidase is an enzyme of the intestinal brush border that is responsible for the digestion of the oligosaccharides into absorbable monosaccharides. The alpha-glucosidase inhibitors are used in diabetes treatment to delay carbohydrate intestinal digestion and reduce glucose uptake [29]. The “stimulating” bioactivity is a very broad category that included any peptide that stimulates biological processes that do not yet have a category of their own. Among the bioactive fragments identified in this study, the fragments were found that stimulate glucose uptake and release of vasoactive substances. 

The DPP-III inhibitory fragments were also found. DPP-III is a peptidase widely distributed in the human body, that is responsible for opioid peptides’ (enkephalins) degradation [30]. The DPP-III inhibitors have therefore excellent therapeutic potential in pain management. DPP-III’s overall physiological role is not yet fully understood, but its role in cancer, oxidative stress defense [30] and blood pressure regulation [31] have been listed.

In the light of these results, the antidiabetic, antihypertensive and antioxidant activities were selected to be further investigated, as these bioactivities are associated with the health benefits of foremost importance.

### 2.3. Characterization of the 3 kDa Permeate of Legume Digestates 

**Figure 4:**
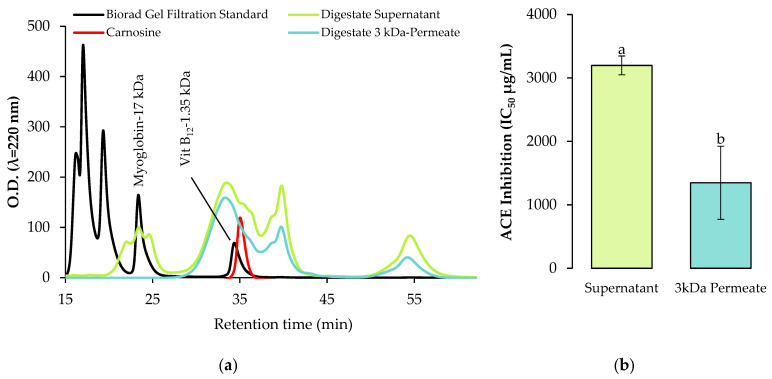
(**a**) Molecular weight distribution of peptides; and (**b**) exhibited antihypertensive activity of the faba bean digestate (variety Fabelle) before and after 3 kDa cut-off ultrafiltration. The ACE inhibition data are expressed as mean ± standard deviation and means without a common letter differ (*p* < 0.05) as analyzed by one-way ANOVA and the Tukey’s test.

In this study, the in vitro gastrointestinal digestates were filtered on a 3 kDa membrane to collect the small molecular weight peptides. It is well established that the peptide bioactivity potency is highly affected by the amino acid chain length [32], and that the peptides with the highest activity are usually composed of 2–20 amino acids [14]. The efficiency of the ultrafiltration step to remove the remaining large soluble proteins was verified by size exclusion HPLC (Figure 4). The size exclusion HPLC analysis of the faba bean (Fabelle variety) digestate before and after the 3 kDa cut-off ultrafiltration demonstrates the removal of the high molecular weight proteins and polypeptides (>17 kDa). These could be the remaining digestive enzymes and the undigested faba bean proteins. Moreover, the antihypertensive activity of the faba bean digestate shows an important increase after ultrafiltration, as demonstrated by the significant decrease (*p* < 0.05) in the ACE inhibition IC_50_ (Figure 4). This indicates that the 3 kDa cut-off ultrafiltration is a valuable approach to recover most of the potent bioactive peptides.

**Figure 5:**
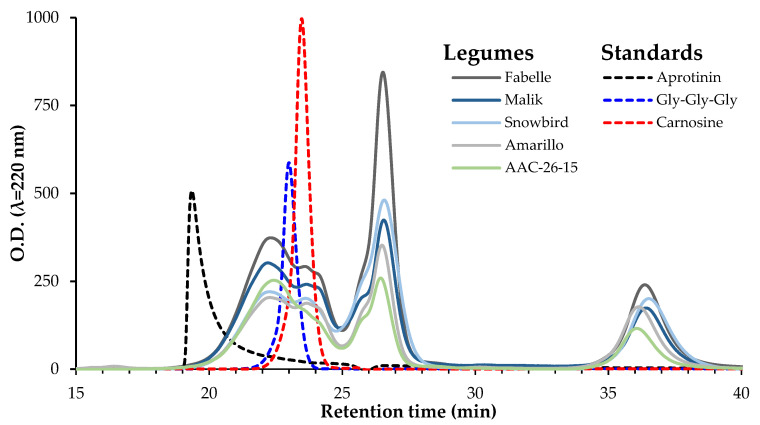
Molecular weight distribution of peptides in the 3 kDa permeate of legume digestates as assessed by size exclusion HPLC.

The size exclusion HPLC profiles of the five legumes’ 3 kDa permeates are shown in Figure 5. There were no noticeable differences among the faba beans, pea and soy profiles. All of the patterns were composed of three main peaks with retention times of ~22.5, 26.5 and 36.5 min. The first peak had a retention time overlapping the carnosine (~226 Da) and Gly-Gly-Gly (~189 Da). This peak is likely to correspond with the small peptides with molecular weights in the same range as these standards. The two other peaks had higher retention times than the dipeptide carnosine standard, therefore probably corresponding to the amino acid residues.

Since the peptides are not the only potential bioactive constituent of the legume digestates found in the targeted molecular weight range (<3 kDa), a composition analysis of the 3 kDa permeates was performed (Table 2). The peptides accounted for 34.4–38.4%, 32.9% and 54.5% of the 3 kDa permeate content for the faba bean, pea and soy, respectively, while the carbohydrates accounted for 43.5–45.2, 46.7 and 16.6 g of the glucose equivalent/g, respectively. Similar concentrations of the total polyphenols, ranging from 4.43 mg to 4.97 mg of gallic acid equivalent/g were found in all of the three studied legumes permeate. The observed differences in the carbohydrate and protein contents among the faba bean, pea, and soy 3 kDa permeate is most probably related to the differences in the seeds’ composition. The polyphenols and carbohydrates also reported bioactive properties [33,34,35,36,37,38], and could therefore also contribute to the exhibited 3 kDa permeates bioactivities.

### 2.4. Bioactive Properties of the 3 kDa Permeate of Legume Digestates 

#### 2.4.1. Antioxidant and Chelating Activities

Various assays, based on different mechanisms of action, were used to assess the antioxidant activity of the 3 kDa permeate of the legume digestates [39,40,41,42]. Indeed, the antioxidants can quench free radicals either through single electron transfer (SET), hydrogen atom transfer (HAT) or a combination of both [43]. The 2,2-diphenyl-1-picrylhydrazyl (DPPH) and the 2,2′-azino-bis(3-ethylbenzothiazoline-6-sulfonic acid) (ABTS) assay are free-radical quenching assays that are mainly SET-based [44,45]. On the other hand, the oxygen radical absorbance capacity (ORAC) assay is HAT-based. The antioxidants can also prevent the free radicals’ formation through the chelation of transition metal ions that act as catalysts in free radicals’ formation, such as the Fenton reaction [46,47]. The iron chelating assay measures this preventive capacity of antioxidants. 

**Figure 6:**
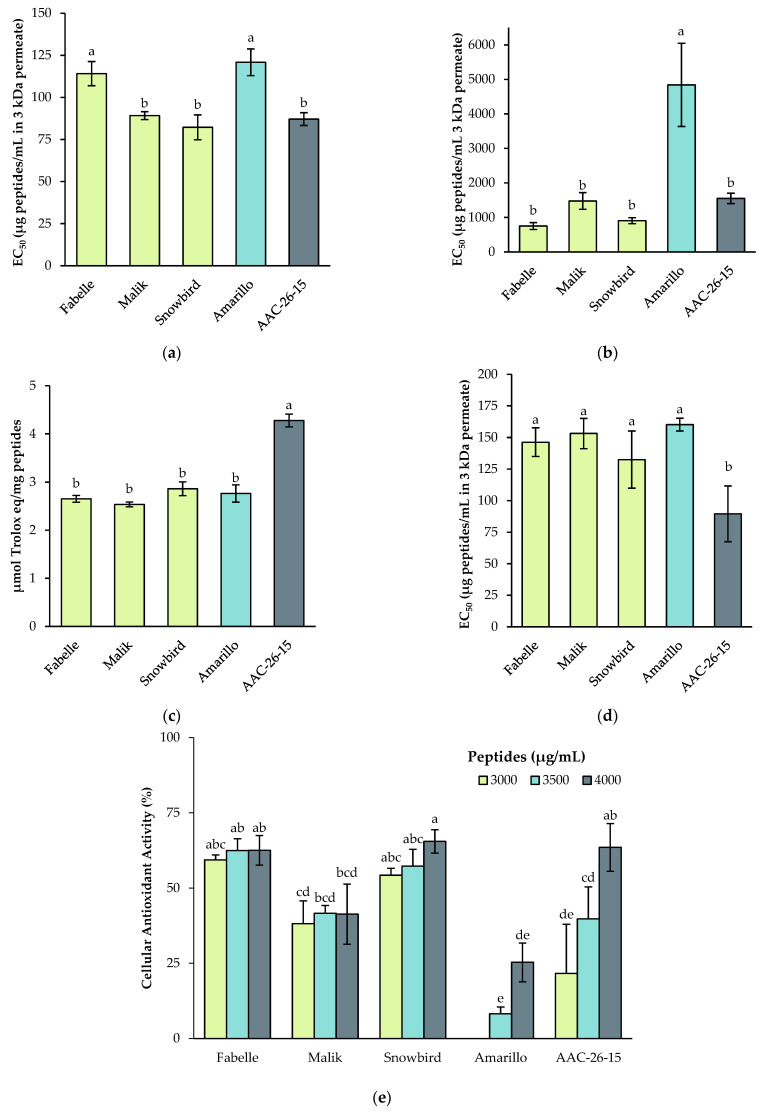
Antioxidant activity of the 3 kDa permeate of legume digestates as assessed by (**a**) the 2,2′-azinobis(3-ethylbenzothiazoline-6-sulfonic acid) (ABTS) assay; (**b**) the 2,2-diphenyl-1-picrylhydrazyl (DPPH) assay; (**c**) the oxygen radical absorbance capacity (ORAC); (**d**) the iron chelating assay; and (**e**) the cellular antioxidant assay (CAA). Data are expressed as mean ± standard deviation and means without a common letter differ (*p* < 0.05) as analyzed by one-way ANOVA and the Tukey’s test.

In the four antioxidant assays conducted in vitro, no significant differences were found among the faba beans varieties (Figure 6), except for the ABTS assay in which the Fabelle variety had a significantly lower activity (*p* < 0.05). Overall, the faba bean varieties had either a lower or similar antioxidant power compared to the soy, and a similar or higher antioxidant power than the pea. In the DPPH assay, the EC_50_ of the peas was significantly higher than the soy and faba beans (*p* <0.05), suggesting a lower antioxidant activity. In the ABTS assay, the same tendency was observed, except for the Fabelle variety, which had a similar EC_50_ than the peas and a higher EC_50_ than the Malik, Snowbird and soy. In the ORAC assay, a different outcome was observed, where the antioxidant activity of the soy was higher compared to the faba beans and peas. Besides, there was a significant correlation between the DPPH and ABTS assay results (r = 0.522; *p* = 0.046), but not with the ORAC assay, which is expected due to its different mechanism of action. For the iron chelating assay, there were no significant differences between the EC_50_ of the faba beans and peas. However, the EC_50_ of the soy was significantly lower (*p* < 0.05), which suggest a higher chelating activity. 

The ABTS EC_50_ value obtained for the studied faba bean varieties were lower than the reported values by Jakubczyk et al. [19] for a 3 kDa permeate of an in vitro gastrointestinal digestate of fermented faba bean seeds. In their study, the EC_50_ value ranged from 990 to 3510 µg/mL, depending on the fermentation conditions used, whereas in this study the EC_50_ varied from 82 to 114 µg/mL. For the iron chelating activity, the EC_50_ obtained by Parya Samaei et al. [48] for the faba bean protein hydrolyzed with Alcalase, pepsin and/or trypsin were lower (EC_50_ = 3.50–8.62 µg/mL) than the levels obtained in this study (EC_50_ = 132–153 µg/mL). The DPPH assay results were in the same order of magnitude as the results obtained by Karkouch et al. [20] for the peptide fractions separated by strong cation-exchange chromatography obtained from a faba bean trypsin hydrolysate. The DPPH scavenging ranged from 40 to 85% when the peptide fractions were tested at 1000 μg/mL whereas, in this study, 50% DPPH scavenging was obtained at concentrations varying from 752 to 1477 μg/mL. Nonetheless, the disparities in the faba bean varieties, sample digestion conditions and antioxidant assay procedures make comparisons difficult between studies.

The antioxidant activity of the 3 kDa permeates was also assessed, using a cellular model to evaluate the antioxidant activity in a more physiologically relevant manner. The cellular antioxidant assay considers diverse mechanisms, including direct free-radical quenching and the stimulation of antioxidant enzyme actions, in experimental conditions that take into account cell uptake and metabolism [49,50]. The Caco-2 cell line was used, which is a well-established model of small intestine enterocytes. At the highest peptide concentration tested (4000 μg/mL), the peas had a lower antioxidant activity compared to the soy and faba beans (*p* < 0.05), as shown by a lower Cellular Antioxidant Activity (CAA) value. Among the faba bean varieties, Snowbird had a significant higher CAA value compared to Malik. For the faba bean varieties, the CAA value was similar for the three concentrations tested, which suggest that these concentrations were in the upper-plateau of the dose–response curve. However, for the soy and pea, the CAA values significantly decreased with concentration and reached no effect in the case of the peas at the lowest dose tested. At the lowest peptide concentration tested (3000 μg/mL), the CAA value was significantly higher for the Fabelle and Snowbird variety compared to the soy and pea. At the same concentration, the CAA value of the Malik variety was similar to the soy but higher than pea. Those results suggest that at lower doses, the antioxidant effect of the faba bean peptides is higher compared to the pea and soy peptides. This outcome is slightly different than the results observed with the in vitro assays, where the soy had overall better antioxidant activity compared to the faba beans. This effect could possibly be explained by a better bioavailability and/or a better resistance to cell metabolism of the faba bean peptides compared to the pea and soy peptides. Further research will be needed to investigate this hypothesis. It is also possible that the faba bean peptides had a better activity in this particular assay. The dissimilarities between in vitro antioxidant assays and CAA results have been observed by Wan et al. [51] as well. They found that the in vitro ORAC and CAA results were not in accordance for numerous purified phytochemicals. However, there was a strong correlation between CAA and in vivo ORAC results (ORAC values of rat plasma after the intake of antioxidants), which state that the CAA result was a better indicator of in vivo antioxidant effect.

To the best of our knowledge, this is the first time that the cellular antioxidant activity of the faba bean hydrolysate have been assessed. More generally, the data relative to the cellular antioxidant activity of a complex food hydrolysate are very limited. Chen et al. [52] have measured the cellular antioxidant activity of the 10-kDa permeate of common bean milk and yogurt in vitro gastrointestinal digestates in Caco-2 cells and have obtained CAA values ranging from 25 to 35%, depending on the doses tested (0.1–0.5 mg/mL). Torres-Fuentes et al. [53] have obtained CAA values ranging from 10 to 30% for chickpea proteins hydrolyzed with pepsin and pancreatin, depending on the dose tested (0.5–5 mg/mL). Zhang et al. [54] investigated the cellular antioxidant activity of the in vitro gastrointestinal digestate of tilapia protein and tilapia protein hydrolysate prepared with Alcalase in HepG2 cells. They obtained CAA values varying from 30 to 50%, depending on the doses tested (0.1–5.0 mg/mL) and the hydrolysate preparation conditions. The CAA values obtained in this study for the Fabelle and Snowbird faba bean varieties (~60%) are higher than the reported values for common bean milk/yogurt and tilapia proteins, while the CAA values for the Malik variety were in the same range (~40%). Nonetheless, the differences in the experimental conditions for the in vitro gastrointestinal digestion, as well as the cell culture conditions, make comparisons quite difficult from one study to another. 

The antioxidant effects of the legume 3 kDa permeates are possibly caused by a synergistic effect of the peptides and other bioactive constituents. For instance, the polyphenols have well-established free radical scavenging and chelating activities [55]. However, our results did not show any significant correlations between the total polyphenol content (TPC)/g of the proteins in the 3 kDa permeate of the legume digestate and the DPPH and ABTS scavenging activity and the CAA values. Moreover, there was a significant inverse correlation between the TPC content/g of proteins and the iron chelating activity (*p* < 0.0001) and the ORAC score (*p* = 0.00037), meaning that the lowest TPC content/g of the proteins in the 3 kDa permeates lead to the highest antioxidant activity. Since the TPC content was similar among the five studied legumes, it can be inferred that the bioactive peptides are possibly responsible in a larger proportion for the observed differences in the antioxidant effect among the samples.

#### 2.4.2. Antihypertensive Activity

**Figure 7:**
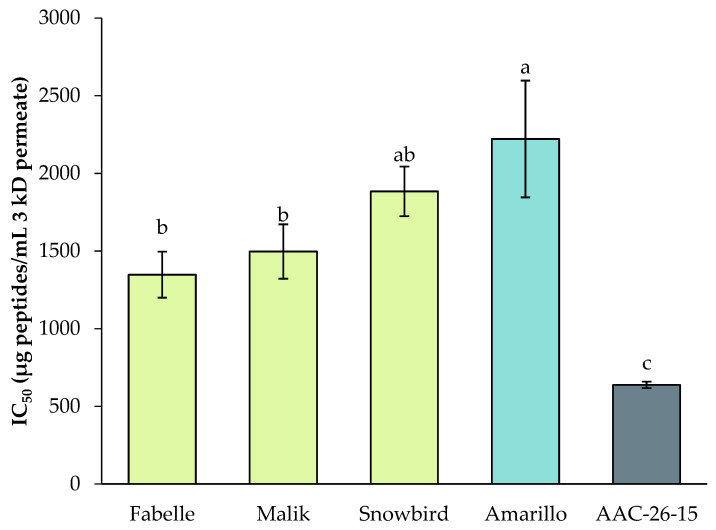
Antihypertensive activity (Angiotensin-Converting Enzyme inhibition) of the 3 kDa permeate of legume digestates. Data are expressed as mean ± standard deviation and means without a common letter differ (*p* < 0.05) as analyzed by one-way ANOVA and the Tukey’s test.

The antihypertensive activity of the legume 3 kDa permeates was evaluated by means of the Angiotensin-Converting Enzyme (ACE) inhibition assay (Figure 7). There were no significant differences for the ACE inhibition activity among the faba bean varieties. The soy had a significantly lower IC_50_ compared to the faba beans and peas, which means a higher antihypertensive activity. The Fabelle and Malik varieties had a significantly higher antihypertensive activity compared to the pea, but the Snowbird variety was similar to the pea. The obtained IC_50_ for the faba beans were in the same range as the values obtained by Jakubczyk et al. [19] for a 3 kDa permeate of an in vitro gastrointestinal digestate of fermented faba bean seeds. In their study, the IC_50_ varied from 1010 to 2920 ug/mL, depending on the fermentation condition used, whereas in this study the values varied from 1348 to 1884 µg/mL. Dugardin et al. [21] obtained a lower IC_50_ value for an in vitro gastrointestinal digestate of faba bean protein isolate (IC_50_ = 52 ug/mL), which can be explained by experimental conditions disparities, such as the lower ACE unit used in their study (0.05 U/mL) compared to ours (0.8 U/mL). Moreover, the faba bean protein isolate could possibly have a higher digestibility rate than the faba bean flour, leading to a higher content of the smaller and highly bioactive peptides.

#### 2.4.3. Antidiabetic Activity

The antidiabetic activity of the legume 3 kDa permeates was assessed, using both in vitro and cellular Dipeptidyl Peptidase-IV (DPP-IV) inhibition assays. DPP-IV is a peptidase with multiple functions in the body and is found in the intestinal enterocytes’ membrane, among others. The Caco-2 cells that express DPP-IV are therefore a relevant model to study the inhibitory potential of the dietary bioactive peptides. In addition, they have the advantage of mimicking the cell metabolism and proteolysis that occur at the brush border, with a cell viability and experimental conditions that are closer to the physiological context [56,57,58,59].

**Figure 8:**
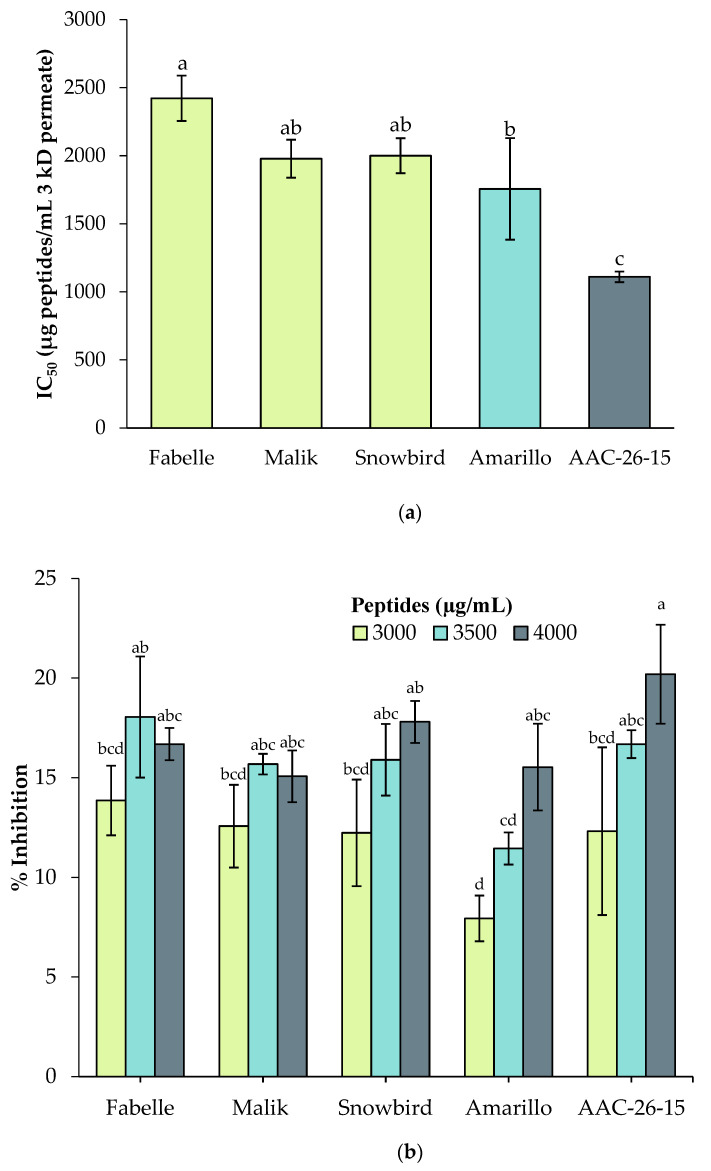
Antidiabetic activity (Dipeptidyl-Peptidase-IV inhibition) of the 3 kDa permeate of legume digestates: (**a**) in vitro assay with a purified Dipeptidyl-Peptidase-IV from porcine kidney; (**b**) inhibition assay in a cellular model. Data are expressed as mean ± standard deviation and means without a common letter differ (*p* < 0.05) as analyzed by one-way ANOVA and the Tukey’s test.

In the in vitro assay, the IC_50_ of the soy was significantly lower compared to the faba beans and peas, which demonstrate a higher antidiabetic potency (Figure 8). This result followed the same trend as the ACE inhibition assay, in which the soy IC_50_ was the lowest compared to the faba beans and peas. There were no significant differences among the faba bean varieties. However, the Fabelle variety had a significantly higher IC_50_ compared to the peas and therefore a lower antidiabetic activity. Dugardin et al. [21] obtained a lower IC_50_ value for an in vitro gastrointestinal digestate of faba bean protein isolate (IC_50_ = 540 ug/mL) compared to this study, where the IC_50_ for the faba beans varied from 1979 to 2400 µg/mL. As above, this variation can be explained by the differences in the sample nature (faba bean flour versus protein isolate) and the digestion conditions. For instance, the gastrointestinal digestion procedure of Dugardin et al. [21] included only a gastric and duodenal digestion phase, whereas ours included an additional jejunal–ileal digestion phase. The addition of this last digestion phase mimics more closely the physiological conditions of protein digestion, and is likely to have an impact on the peptide profile found in the digestate [60].

In the cell model (Figure 8), there were no significant differences among the faba beans, peas and soy. However, as observed in the Cellular Antioxidant Assay, there was a dose–response effect observed for peas and soy; the DPP-IV inhibition effect was significantly lower at 3000 µg/mL compared to 4000 µg/mL, and there were no significant differences among the studied faba bean protein concentrations. Again, this effect may be explained by a better bioavailability and/or better resistance to the brush border peptidase degradation of the faba bean peptides compared to the pea and soy peptides. This hypothesis deserves further investigation. 

Nonetheless, the percentage of DPP-IV activity inhibition in the cell model remains lower compared to the in vitro results. In the cell-based assay, the percentage of activity inhibition ranged from 15 to 20% at 4000 µg/mL, versus 68 to 90% in the in vitro assay at the same concentration. The same trend was observed by Caron et al. [56], where the IC_50_ of a bovine hemoglobin gastrointestinal digestate was 10 times higher in the cell model (16.02 mg/mL) compared to the in vitro assay (1.62 mg/mL), meaning that the activity was ten times lower in the cell assay compared to the in vitro assay. Aiello et al. [59] also found the same tendency for a spirulina protein hydrolysate. Lacroix and Li-Chan [61] demonstrated that porcine DPP-IV was more easily inhibited by the peptides as compared to the human DPP-IV, which can explain this finding. This observation may also be explained by a further hydrolysis of the bioactive peptides once incubated with Caco-2 cells, which express numerous peptidases of the intestinal brush border. This finding reasserts the importance of investigating the bioactive properties with cell models and not exclusively with in vitro assays, to obtain a more realistic picture of the potential bioactivities in vivo. 

### 2.5. Peptides Fractionation and Sequencing

**Figure 9:**
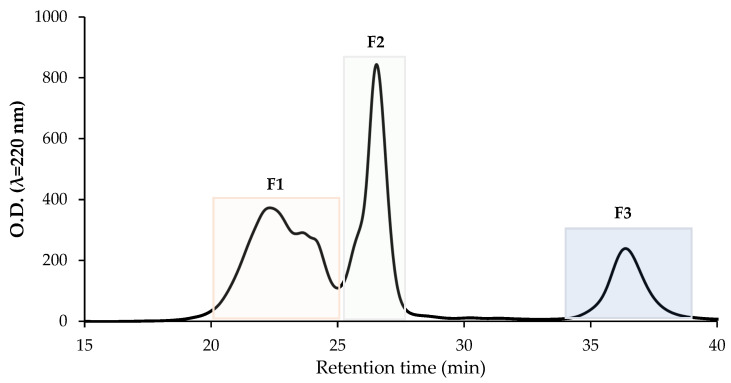
Peptide fractionation of the 3 kDa permeate of Fabelle in vitro gastrointestinal digestate by size exclusion HPLC. F1, fraction 1; F2, fraction 2; F3, fraction 3.

**Figure 10:**
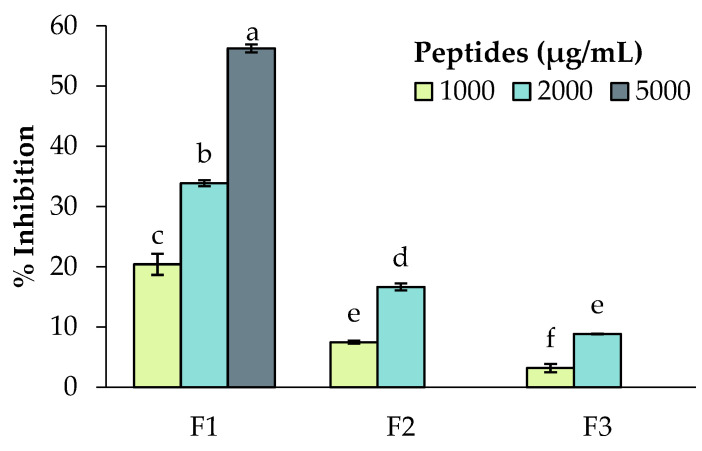
Angiotensin-Converting Enzyme inhibition of faba bean (variety Fabelle) peptide-enriched fractions (F1, F2 and F3). Data are expressed as mean ± standard deviation and means without a common letter differ (*p* < 0.05) as analyzed by one-way ANOVA and the Tukey’s test.

Based on the results of the in vitro and cellular bioactivity assays, the Fabelle variety was selected to be investigated further, since it stood out for its antihypertensive and antioxidant activities. Moreover, this new variety contains a low amount of the anti-nutrients vicine, convicine and tannins, which represents an important advantage for food applications. The Fabelle 3 kDa permeate was fractionated by size exclusion HPLC and three peptide fractions were recovered (Figure 9). The three collected fractions were tested again for their antioxidant and antihypertensive activities. The results indicated that F1 had the highest antihypertensive potency, followed by F2 and F3 (Figure 10). F2 had the highest free radical scavenging activity through SET, as demonstrated by the results of the ABTS assay, while F2 and F3 were equal for their free radical scavenging activity through HAT, as indicated by the results of the ORAC assay (Table 3). The F3 was the fraction with the highest iron chelating activity (Table 3).

Interestingly, the fractionation caused either a loss or an increase in the bioactive activities compared to the unfractionated 3 kDa permeates. In the case of the ACE inhibition, a 50% inhibition was obtained at 1348 μg/mL for the unfractionated Fabelle 3 kDa permeate, whereas the inhibition varied from 8 to 34% for the fractions at 2000 μg/mL. It is possible that the peptides present in the different fractions had a synergistic effect on ACE, which explains the activity loss at the fractions’ level. It is also possible that the fractionation removed the other bioactive constituents that contributed to the overall effect against ACE activity. On the contrary, the ABTS scavenging, and iron chelating activities highly increased after fractionation. The same tendency was observed by Jakubczyk et al. [19]; in their study, the EC_50_ for the ABTS scavenging was 0.99 mg/mL before fractionation and decreased to 0.02–0.1 mg/mL after fractionation. The removal of the other constituent of the 3 kDa permeate with antagonist effect could explain this outcome. In the case of ORAC, F2 and F3 had a higher activity compared to the complete 3 kDa permeate, but F1 had a lower activity, suggesting that the peptides responsible for this antioxidant effect were mostly eluted in F2 and F3.

In order to obtain a better understanding of the observed differences in the bioactive properties of the three fractions, the peptide profile of each fraction was identified by LC-MS/MS and database searching (Table 4). Eleven unique peptides were identified, 11 in F1, 1 in F2 and none in F3. One peptide was present in both F1 and F2, as presented in Table 4. This is most probably due to some peak overlapping during the size exclusion chromatography, as previously observed by Torres-Fuentes et al. [53]. Most of the peptides were found in F1, which is in accordance with the determined protein content of each fraction. Approximately 70% of the initial proteins of the 3 kDa permeate were recovered in F1, 20% in F2 and 10% in F3. The peptides were composed of 9 to 11 amino acid residues, with molecular weights ranging from 888 Da to 1336 Da, which is in the typical range of highly active peptides [14]. Seven identified peptides were from the globulin storage proteins (legumin, vicilin and convicilin). Those peptides are likely to contribute significantly to the faba bean protein bioactive properties, since the globulins account for up to 80% of the faba bean seed proteins [62].

The relationship between the peptide chemical structure and bioactivity is not yet well understood [63,64]. However, the peptide length, charge, amino acid composition and the particular order and the presence of the hydrophobic residue are all of the factors influencing the bioactivity potency [20,65]. The potential bioactive properties of the LC-MS/MS identified peptides were screened in silico, using the BIOPEP-UWM database and the results are presented in Table 5. All of the identified peptides contained fragments with an inhibitory effect against ACE and DPP-IV, and four of them also had antioxidant fragments, which corroborated our experimental findings. The results (Table 5) revealed that the peptide PVNRPGEPQ has a very promising bioactive potential, especially for ACE and DPP-IV inhibition. Indeed, the fragments with these specific activities are present in high frequency and those fragments are highly active, as revealed by the high B parameter values. Noteworthy, this peptide was found in F1, which was the fraction with the highest measured ACE inhibitory activity. The peptides VIPTEPPH, VIPTEPPHA, VVIPTEPPH and VVIPTEPPHA contains as well a high frequency of ACE and DPP-IV inhibitor fragments, however, their predicted activity is lower. The peptides EEEDEDEPR and KEEEDEDEPR contain a low frequency of ACE inhibitor fragments, but have a high predicted activity.

The peptide TETWNPNHPEL has the highest antioxidant fragment frequency among the identified peptides. Indeed, this peptide was previously identified by Torres-Fuentes et al. [53] in a pepsin-pancreatin chickpea protein hydrolysate for its antioxidant and chelating activity. The finding of the same peptide in the present study suggests that the *Fabaceae* storage proteins are highly conserved. Although the F2 and F3 had the highest measured antioxidant activity overall, only one peptide was successfully identified and no in silico predicted antioxidant activity was associated with it. Still, this peptide contains a histidine, glutamic acid and threonine residue that are well known for their contribution to the iron-chelating activity [66]. Histidine is also implicated in free radical scavenging [66]. The peptides identified in F2 and F3 remain very limited. The faba bean proteome is incomplete which makes the peptide identification challenging. It is also possible that F2 and F3 contained very low-molecular weight peptides and the free amino acids that were not detected. Still, the chelating activity and ABTS scavenging activity of F1 was importantly higher than the complete 3 kDa permeate, which indicate an excellent antioxidant activity that can be attributed to the peptides.

Other bioactivities were also identified, namely the anti-amnestic, antithrombotic, regulating stomach mucosal membrane activity, DPP-III inhibitor, renin inhibitor, alpha-glucosidase inhibitor and stimulating activities. Noteworthy, most of these activities were identified as well in the in silico screening of the faba bean storage proteins. The alpha-glucosidase inhibition activity of the faba bean ethanol extract [67], and the germinated and fermented faba beans in vitro gastrointestinal digestates [68] were also reported by others, which is in agreement with our finding. The other identified activities, however, were not so far experimentally investigated in faba beans.

## 3. Materials and Methods

### 3.1. Materials 

Three dehulled faba bean cultivars (Fabelle, Malik and Snowbird), one dehulled pea cultivar (Amarillo) and one dehulled soy cultivar (AAC-26–15) were used in this study. The faba bean and pea samples were supplied as milled flours, and soybean as whole seeds. The faba bean cultivars, Fabelle and Malik, were provided by AGT Foods and Ingredients (Saskatoon, SK, Canada), and Snowbird by W.A. Grain & Pulse Solutions (Innisfail, AB, Canada). The certified yellow pea (CDC Amarillo) and soybean (Cdn #1, Variety AAC 26-25, Non-GMO and IP, Lot 261510504AT) were provided by Greenleaf Seeds (Tisdale, SK, Canada) and Huron seeds (Clinton, ON, Canada), respectively.

The hexane, sulfuric acid, dimethyl sulfoxide (DMSO) and methanol were obtained from Fisher Scientific (Fair Lawn, NJ, USA). The ethanol was purchased from Commercial Alcohols (Brampton, ON, Canada).

The hydrochloric acid (HCl), sodium hydroxide (NaOH), Glucose, 4-(2-aminoethyl)benzenesulfonyl fluoride hydrochloride (AEBSF), Phosphate-buffered saline (PBS), sodium bicarbonate (NaHCO_3_), monosodium phosphate (NaH_2_PO_4_), hydrochloric acid (HCl), sodium chloride (NaCl) and Tris-HCl were purchased from BioShop (Burlington, ON, Canada). 

The 2,2′Azobis (2-methylpropionamidine) dihydrochloride (AAPH), Trolox, fluorescein, 2′-7′-Dichlorofluorescein diacetate (DCFH-DA), Gly-Pro-7-amido-4-methylcoumarin hydrobromide (Gly-Pro-AMC), Dipeptidyl Peptidase IV (CD26) from Porcine Kidney, 2,2-diphenyl-1-picrylhydrazyl (DPPH), 2,2′-azino-bis(3-ethylbenzothiazoline-6-sulfonic acid) diammonium salt (ABTS), potassium persulfate (K_2_S_2_O_8_), ferrous chloride (FeCl_2_), ferrozine, borax (Na_2_B_4_O_7_·10H_2_O), angiotensin-converting enzyme (ACE) from rabbit lung (A6778), N-Hippuryl—His—Leu hydrate (HHL) substrate (H1635), trifluoroacetic acid (TFA), Gallic Acid, Folin–Ciocalteu and phenol were purchased from Sigma-Aldrich (St. Louis, MO, USA).

For the in vitro digestion procedure, α-amylase from porcine pancreas (A3176), pepsin from porcine gastric mucosa (P6887), pancreatin from porcine pancreas (P7545), trypsin from porcine pancreas (T0303), α-chymotrypsin from bovine pancreas (C7762), porcine bile extract (B8631) and the Bile Acid Assay Kit (MAK309) were all purchased from Sigma-Aldrich (St. Louis, MO, USA). The native Porcine Peptidase (Nate-0548) was purchased from Creative Enzyme (Shirley, NY, USA). 

For the cell culture, Dulbecco’s Modification of Eagle’s Medium (DMEM) containing 4.5 g/L glucose, with phenol-red and without sodium pyruvate, 200 mM L-Glutamine, Dulbecco’s Phosphate-Buffered-Saline (D-PBS) without Ca^2+^ and Mg^2+^, Nonessential Amino Acid Solution 100×, heat-inactivated fetal bovine serum (FBS), 5000 IU penicillin and 5000 μg/mL streptomycin solution, Trypsin solution (0.05%) containing 0.53 mM EDTA in HBSS and Trypan blue (0.4%) were purchased from Wisent Bioproducts (Saint-Jean-Baptiste, QC, Canada). Hank’s Balanced Salt Solution (HBSS) was purchased from Gibco (Thermo Fisher Scientific, San Jose, CA, USA). The Caco-2 cells (ATCC^®^ HTB-37™, passage 18) were purchased from ATCC (Manassas, VA, USA).

The Pierce BCA Assay kit was purchased from Thermo Fisher Scientific (San Jose, CA, USA) and the Cell Titer-Glo 2.0 kit was purchased from Promega (Fitchburg, WI, USA). 

All of the chemicals and reagents used were of analytical grade and deionized water (Millipore) was used in all of the experiments. 

### 3.2. Sample Preparation

The soy seeds were milled with a Brinkmann centrifugal grinding mill using a 0.2-mm rotary sieve with the addition of liquid nitrogen to prevent heating. The soy flour was then de-oiled through hexane extraction, according to the method of L’Hocine et al. [71] to obtain a fat content similar to the faba beans and peas. The faba bean, pea and soy flours were cooked (thermally treated through boiling), which is a representative domestic processing for legume preparation, following the procedure of Ma et al. [72]. The flour was hydrated in water (ratio 1:10) for 1 h at room temperature under constant stirring and then boiled for 20 min. The cooked flour and cooking water were frozen at −40 °C, freeze-dried and milled once more to assure a particle size uniformity among the samples. All of the flour samples were stored at −20 °C in vacuum bags until needed.

### 3.3. Adaptation of the In Vitro Gastrointestinal Digestion Procedure for Cell Culture 

The gastrointestinal digestion was simulated using the INFOGEST harmonized static in vitro digestion procedure [25] with some modifications. Indeed, Brodkorb et al. [25] suggested investigating whether the bile salts and protease inhibitor concentration interfere with the cell model, since those two elements can compromise cell viability. The preliminary studies were conducted to investigate the impact of those elements on the specific cell line used in this work, the Caco-2 cells. Three parameters were considered: the enzyme used during the intestinal digestion phase (Pancreatin or a mix of trypsin (100 U/mL digest); α-chymotrypsin (25 U/mL digest) and α-amylase (200 U/mL digest), with or without the addition of a jejunal-ileal digestion phase), the bile salts’ concentration (1 or 10 mM) and the protease inhibitor (4-(2-Aminoethyl) benzenesulfonyl fluoride hydrochloride or AEBSF) concentration (0.25, 1 or 5 mM). The digestions were performed with one faba bean variety (Fabelle) in triplicate and one parameter was varied at the time.

### 3.4. In Vitro Gastrointestinal Digestion of Legume Flours 

Once the adjusted digestion conditions for the cell culture were obtained, the in vitro gastrointestinal digestate of the different legume flours was prepared accordingly. Prior to digestion work, the enzyme activity of amylase, pepsin, pancreatin, trypsin and chymotrypsin were determined, according to Brodkorb et al. [25] procedures and the bile salts content in the porcine bile extract was assessed, using the Sigma Bile Assay Kit (MAK309). The leucine aminopeptidase activity in the porcine intestinal peptidase was evaluated, following the procedure of Hausch et al. [73] and one unit was defined as the consumption of 1 μmol of leucine-*p*-nitroanilide per minute at 30 °C and pH 8.0. 

Briefly, 1.2 g of flour was mixed with 1.8 g of water to reach the targeted consistency of tomato paste at the end of the oral phase. For the oral digestion phase, the hydrated flour was mixed in a ratio 1:1 with simulated salivary fluid (SSF) containing α-amylase from the porcine pancreas (75 U/mL digestate) and digestion was conducted for 2 min at 37 °C under constant stirring. For the gastric digestion phase, the digestate was mixed in a ratio 1:1 with simulated gastric fluid (SGF), containing pepsin (2000 U/mL digestate). The pH was adjusted to 3.0 by the dropwise addition of 6 N HCl, and the digestion was continued for 2 h at 37 °C under constant stirring. The gastric lipase was not added since lipid is a minor constituent in the sample studied (<1%) compared to the proteins and starch. The digestates were diluted once more for the duodenal digestion phase, in a ratio 1:1 with the Simulated Intestinal Fluid (SIF) containing pancreatin from the porcine mucosa (100 U trypsin activity/mL digestate) and the porcine bile extract (10 mM bile salts), the pH was adjusted to 7.0 with 3 N NaOH and the digestate was incubated for 2 h at 37 °C with constant stirring. To simulate the digestion that occurs at the brush border in the jejunum and ileum, the pH was adjusted to 7.2 and a Native Porcine Peptidase (13 mU leucine-aminopeptidase activity/mL digestate) was added. The digestate was incubated for another 4 h at 37 °C under constant stirring.

At the end of the digestion, the samples were cooled on ice and the protease inhibitor (1 mM AEBSF) was added to stop the digestion. The digestates were centrifuged at 15,000× *g* for 30 min at 4 °C and the supernatants were filtered using an Amicon ultrafiltration system equipped with a 3 kDa molecular weight cut-off (MWCO) regenerated cellulose membrane (END Millipore, Billerica, MA, USA) to recover the small molecular weight peptides. The peptide stability during the ultrafiltration step was verified in quantifying the free amino groups using the 2,4,6-Trinitrobenzene Sulfonic Acid (TNBS) method of Adler-Nissen [74], with modifications by Mason [75]. 

The permeates’ osmolality was measured and the permeates were diluted in water to reach a physiological osmolality of 285–300 mOsm/kg, using a Micro-Osmometer (Model 3320; Advanced Instruments Inc., Norwood, MA, USA). Their pH was also adjusted to a physiological value of 7.3. The diluted 3 kDa permeates were analyzed for their protein content with the Pierce Bicinchoninic Acid (BCA) protein assay kit, using bovine serum albumin as standard and frozen at −80 °C until further use. A portion of the frozen permeates was freeze-dried and stored at −20 °C until further characterization. 

### 3.5. In Silico Prediction of Bioactive Fragments Released during Gastrointestinal Digestion of Legume Proteins

The potential bioactive fragments released during the gastrointestinal digestion of the major faba bean, pea and soy storage proteins were investigated using an in silico analysis. The accession numbers of the protein used for the analysis are displayed in Table 6. The globulins’ sequences were hydrolyzed in silico with pepsin (pH > 2) (EC 3.4.23.1), trypsin (EC 3.4.21.4) and α-chymotrypsin (EC 3.4.21.1) simultaneously to simulate gastrointestinal digestion with the Bioactive Peptide Database of University of Warmia and Mazury (BIOPEP-UWM) enzyme tools [69]. The frequency of the peptide fragments’ release with the selected enzyme activity values (A_E_) for each of the selected protein and bioactivities were automatically calculated by the BIOPEP-UWM algorithm [69] and retrieved on 14 July 2022. 

### 3.6. Characterization of the 3 kDa Permeate of Legume Digestates 

#### 3.6.1. Molecular Weight Distribution of Peptides by Size Exclusion HPLC

The molecular weight distribution profile of the peptides present in the 3 kDa permeate of legume digestate was determined by size exclusion chromatography, following the procedure of Achouri et al. [76] with modifications by Mason et al. [77]. An Agilent-1200 Series HPLC system (Agilent Technologies Canada, Inc., Mississauga, ON, USA), equipped with a Enrich SEC-70 column (10 × 300 mm) (Bio-Rad Laboratories, Mississauga, ON, USA), was used. The samples from the 3 kDa permeate of legume digestate (2.5 µL) were loaded on the column and eluted with 10-mM phosphate buffered saline, with 154-mM NaCl (pH 7.4) at a flow rate of 0.5 mL/min. The absorbance was monitored at λ = 220 nm. The standards, including aprotinin (6.511 kDa), vitamin B12 (1.355 kDa), Gly-Gly-Gly (tripeptide, 189 Da) and carnosine (dipeptide, 226 Da), were used to evaluate the samples’ molecular weight distribution.

#### 3.6.2. Composition Analysis of the 3 kDa Permeate of Legume Digestates

The protein content in the 3 kDa permeate of legume digestate was determined according to the Dumas method [78] with a Vario MAX Cube (Elementar, Langenselbold, Germany) using a nitrogen to protein conversion factor of 6.25 and EDTA as standard. 

The total phenolic content was determined by the Folin–Ciocalteu method following the procedure of Singleton et al. [79] with modifications. A total of 50 mg of freeze-dried permeate was extracted in 1 mL of 70% ethanol containing 1% (*v/v*) 12 N HCl, in the dark, at room temperature and under constant stirring for 2 h. The samples were then centrifuged (16,000× *g* for 15 min) and filtrated on 0.45 μm Polyethersulfone (PES) filter (Canadian Life Science, Peterborough, ON, USA). The gallic acid standard (50–500 mg/L) was prepared in the same solvent. A total of 200 μL of the samples and standard were mixed with 1.5 mL of Folin–Ciocalteu reagent (diluted 1:10 in water) and incubated in the dark for 5 min. Then, 1.5 mL of 7.5% (*m/v*) sodium bicarbonate solution was added, and the samples were incubated for 75 min in the dark at room temperature. The samples were centrifuged (6000× *g* for 15 min) and the absorbance was read at λ = 750 nm. The total phenolics content (TPC) was expressed as mg of gallic acid equivalent per g of 3 kDa permeate of legume digestates (on a dry basis).

The total carbohydrate content was determined, following the method of DuBois et al. [80]. The freeze-dried permeates were solubilized in water (10 mg/mL). A total of 2 mL of each sample and standard (glucose 0–50 mg/L) were added to a glass test tube in duplicate. A total of 50 μL of phenol 80% (m/m) was added to each sample and standard, followed by 5 mL of sulfuric acid 95.5%. The samples were incubated at room temperature for 10 min and then incubated in a water bath at 25 °C for 10 min. The samples were cooled to room temperature and the absorbance was recorded at λ = 490 nm. The results were expressed as g of glucose equivalent per 100 g of freeze-dried 3 kDa permeates.

### 3.7. In Vitro Antioxidant and Chelating Activities of the 3 kDa Permeate of Legume Digestates

#### 3.7.1. 2,2-diphenyl-1-picrylhydrazyl (DPPH) Free Radical Scavenging Assay

The DPPH assay was performed following the method of Orona-Tamayo et al. [81], as described in Mason et al. [77] with slight modifications. A total of 100 μL of diluted 3 kDa permeate of the legume digestate (100–6000 µg peptides/mL) was mixed with 100 μL of 0.2 mM DPPH (prepared in ethanol 100% and stored at −20 °C) in a clear 96-well clear microplate and incubated for 30 min at 37 °C in the dark. The absorbance was read at λ = 517 nm with an Epoch microplate spectrophotometer (Bio-Tek, Winooski, VT, USA). The free-radical scavenging capacity was calculated as follows, after background subtraction:

$$\mathrm{DPPH}\;\mathrm{radical}\;\mathrm{scavenging}\;\mathrm{capacity}\;\left(\%\right)=1-\frac{A_{\mathrm{sample}}}{A_{\mathrm{control}}}$$

where *A_sample_* is the absorbance of the sample and *A_control_* is the absorbance of DPPH in absence of antioxidants. The EC_50_ value was reported, which was defined as the required peptide concentration to scavenge 50% of the DPPH free radicals. The EC_50_ was calculated using a non-linear regression with a four-parameter logistic (4PL) curve of the DPPH free-radical scavenging capacity plotted against its respective peptide concentration.

#### 3.7.2. 2,2′-azinobis(3-ethylbenzothiazoline-6-sulfonic acid) (ABTS) Scavenging Assay

The ABTS assay was performed following the method of Re et al. [41], as described in Mason et al. [77]. The day before the assay, a solution containing 7 mM ABTS and 2.45 mM of potassium persulfate was prepared in water and incubated overnight at room temperature in the dark to generate the free radicals. The next day, the solution was diluted in ethanol 100% to reach an absorbance value of 0.70 ± 0.02 at λ = 734 nm. A total of 50 μL of the 3 kDa permeate of legume digestates (10–400 µg peptides/mL) was mixed with 180 μL of the ABTS solution in a 96well clear flat-bottom plate, and incubated for 6 min at room temperature in the dark. The absorbance was read at λ = 734 nm with an Epoch microplate spectrophotometer (Bio-Tek, Winooski, VT, USA) microplate reader. The free-radical scavenging capacity was calculated as follows, after background subtraction:

$$ABTS\;\mathrm{radical}\;\mathrm{scavenging}\;\mathrm{capacity}\;\left(\%\right)=\left(1-\frac{A_{sample}}{A_{control}}\right)\times 100$$

where *A_sample_* is the absorbance of the sample and *A_control_* is the absorbance of ABTS in the absence of antioxidant. The EC_50_ value was reported, which was defined as the required peptide concentration to scavenge 50% of the ABTS free radicals. The EC_50_ was calculated using a non-linear regression with a 4PL curve of the ABTS free-radical scavenging capacity, plotted against its respective peptide concentration.

#### 3.7.3. Oxygen Radical Absorption Capacity (ORAC) Assay

The ORAC assay was performed following the method of Tomer et al. [82], as described in Mason et al. [77]. All of the solutions were prepared in a 75 mM phosphate buffer pH 7.4. A total of 25 µL of appropriately diluted 3 kDa permeate of legume digestates and Trolox standard (6.25–50 µM) were loaded in a 96-well black plate with a clear bottom. The outermost wells of the 96-well plates were not used to prevent the plate-edge effects. A total of 150 µL of 96 nM fluorescein solution was added to each well and the plate was incubated for 30 min at 37 °C. After the incubation period, 25 µL of 79.65 mM 2,’2-azobis(2-amidinopropane) dihydrochloride (AAPH), a free radical initiator, was added to each well using an automatic injector and the fluorescence was recorded every minute for 90 min (λ_excitation_ = 485 nm, λ_emission_ = 520 nm), using a Synergy HTX microplate reader (Bio-Tek, Winooski, VT, USA). The area under the curve (*AUC*) was calculated for the samples, standards and blanks with the Gen5 Data Analysis Software (BioTek Instruments, Inc., Winooski, VT, USA), using the following regression equation:

$$AUC=1+\frac{RFU_{1}}{RFU_{0}}+\frac{RFU_{2}}{RFU_{0}}+\frac{RFU_{3}}{RFU_{0}}+\ldots \frac{RFU_{90}}{RFU_{0}}$$

where *RFU*_0_ is the initial fluorescence and *RFU_x_* is the relative fluorescence at each time points. Then, the net *AUC* was calculated as follows:

$$\mathrm{Net}\;AUC=AUC_{standards/samples}-AUC_{blank}$$

The net *AUC* value of the Trolox standards was used to build a standard curve. The antioxidant capacity of the samples was expressed as μmol of Trolox equivalent per mg of proteins.

#### 3.7.4. Iron Chelating Activity

The iron-chelating activity assay was performed following the procedure of Orona-Tamayo et al. [81], as described in Mason et al. [77]. A total of 50 µL of diluted 3 kDa permeate (25–1000 µg peptides/mL) of the legume digestates was mixed with 25 µL of 0.25 mM ferrous chloride and 25 uL of 0.625 mM ferrozine in a clear 96-well plate. The plate was incubated for 10 min in the dark at room temperature and the absorbance was recorded at λ = 562 nm. The percentage of iron chelating was calculated as follows:

$$\mathrm{Iron}\;\mathrm{Chelating}\;\left(\%\right)=\left(1-\frac{A_{sample}}{A_{control}}\right)\times 100$$

where *A_sample_* is the absorbance of the sample and *A_control_* is the absorbance of the iron-ferrozine complex in the absence of chelating peptides. The EC_50_ value was reported, which is defined as the required peptide concentration to chelate 50% of the ferrous ions. The EC_50_ was calculated using a non-linear regression, with a 4PL curve of the iron chelating plotted against its respective peptide concentration.

### 3.8. In Vitro Antihypertensive Activity (Angiotensin-Converting Enzyme Inhibition Activity)

The antihypertensive activity was evaluated using the Angiotensin-Converting Enzyme (ACE) inhibition assay of Barbana and Boye [83], as described in Mason et al. [77] with slight modifications. All of the solutions were prepared in a 1 mM borate buffer pH 8.3 containing 0.3 M NaCl. A total of 10 μL of appropriately diluted 3 kDa permeate of legume digestates (100–6000 µg peptides/mL) was mixed with 10 μL of ACE (8 mU; ACE from rabbit lung, A6778; Sigma-Aldrich, St. Louis, MO, USA) in a test tube and incubated for 10 min at 37 °C. Then, 50 μL of 1 mM Hippury-L-histidyl-L-Leucine (HHL) was added and the incubation was continued for 30 min. At the end of the incubation period, 85 μL of HCL 1N par was added to stop the reaction. The reaction mixture was analyzed by reverse-phase HPLC, using a 4.60 × 250 mm Aqua C18 column (5-µm pore size 125 Å; Phenomenex, Torrance, CA, USA). The samples were eluted with 50% (v/v) methanol in water containing 0.1% TFA at a flow rate of 0.5 mL/min for 15 min. The absorbance was monitored at λ = 228 nm. The peak area of HHL was recorded and the *ACE inhibition* was calculated, as follows:

$$\mathrm{ACE}\;\mathrm{Inhibition}\;\left(\%\right)=\left(1-\frac{\left(PA_{a}-PA_{b}\right)}{\left(PA_{a}-PA_{c}\right)}\right)\times 100$$

where *PA_a_* is the peak area of the control (HHL alone, corresponding to 100% inhibition); *PA_b_* is the peak area of the sample (HHL, ACE and inhibitory peptides) and *PA_c_* is the peak area of the reaction blank (HHL and ACE, corresponding to 0% inhibition). The IC_50_ value was reported, which is defined as the required peptide concentration to inhibit 50% of ACE activity. The IC_50_ was calculated using a non-linear regression with a 4PL curve of the ACE inhibition (%) plotted against its respective peptide concentration.

### 3.9. In Vitro Antidiabetic Activity (Dipeptidyl Peptidase-IV Inhibition Assay) 

The in vitro Dipeptidyl Peptidase-IV (DPP-IV) inhibition assay was performed following the procedure of Caron et al. [56], with slight modifications. Then, 50 μL of diluted 3 kDa permeate of legume digestate (100–6000 µg peptides/mL) was mixed with 25 μL of Dipeptidyl Peptidase IV (CD26) from Porcine Kidney (0.018 U/mL) and 50 μL of 0.1 M Tris-HCl buffer pH 8.0 in a 96-well black plate with a clear bottom and incubated at 37 °C for 5 min. Then, 50 μL of Gly-Pro-7-amido-4-methylcoumarin hydrobromide (Gly-Pro-AMC) 1 mM was added and the fluorescence was recorded at 37 °C after 30 min (λ_ex_ = 350 λ_em_ = 450), using a Synergy HTX plate reader (Bio-Tek, Winooski, VT, USA). The peptides, enzymes and substrates were all diluted in a 0.1 M Tris-HCl buffer at pH 8.0. The *DPP-IV inhibition* was calculated as follows, after background subtraction:

$$\mathrm{DPP}-\mathrm{IV}\;\mathrm{Inhibition}\;\left(\%\right)=\left(1-\frac{RFU_{sample}}{RFU_{control}}\right)\times 100$$

where *RFU_sample_* is the fluorescence of the samples and *RFU_control_* is the fluorescence of the control (DPP-IV with a buffer instead of inhibitory peptides). The IC_50_ value was reported, which is defined as the required peptide concentration to inhibit 50% of the DPP-IV activity. The IC_50_ was calculated using non-linear regression with a 4PL curve of the DPP-IV inhibition (%) against its respective peptide concentration.

### 3.10. Cell Culture

The Caco-2 cells were cultivated in growth medium, which was composed of Dulbecco’s Modified Eagle Medium (DMEM) supplemented with 10% heat-inactivated fetal bovine serum (FBS), 100 U/mL penicillin and 100 μg/mL streptomycin, 1% non-essential amino acids and 2 mM L-glutamine and incubated at 37 °C in an atmosphere containing 5% CO_2_. The cells were sub-cultivated once a week at 80–90% confluence using a trypsin–EDTA solution and the culture medium was changed every 2–3 days. The cells were subcultured three times prior to bioactivity assessment to enable cell phenotype stabilization (Hubatsch et al., 2007). The cells between passage 22 and 32 were used in this study. In every assay, the outermost wells of the 96-well plates were not used to prevent plate-edge effects.

### 3.11. Cell Viability 

The cell viability following incubation with a different concentration of 3 kDa permeate of each legume digestate (3000, 3500 and 4000 µg peptide/mL) was verified using the Cell Titer-Glo 2.0 kit (Promega, Fitchburg, WI, USA), which is a luminescent based method that quantify ATP as an indicator of cell viability. The cells were seeded in growth medium in a 96-well black plate with a clear bottom at a density of 6.5 × 10^4^ cells/well for 48 h to reach confluence. On the day of the assay, the medium was discarded, the cells were washed with 100 μL of Dulbecco’s Phosphate-Buffered-Saline (D-PBS) and then incubated with 100 μL of 3 kDa permeate of the legume digestates diluted in DMEM for 1 h at 37 °C. The DMEM was used as a negative control and the H_2_O_2_ (20%) as a positive control. After the incubation period, the plate was equilibrated at room temperature for 30 min and 100 μL of Cell Titer-Glo® 2.0 Reagent (equilibrated previously at room temperature) was added per well. The plate was shaken for 2 min to provoke cell lysis, then incubated 10 min at 22 °C and the luminescence (L) was recorded using a Synergy HTX plate reader (Bio-Tek, Winooski, VT, USA). The cell viability was expressed as follows, after background subtraction:

$$\mathrm{Cell}\;\mathrm{viability}\;\left(\%\right)=\left(\frac{L_{sample}}{L_{control}}\right)\times 100$$

where *L_sample_* is the luminescence of the sample and *L_control_* is the luminescence of the cells incubated in DMEM (corresponding to 100% viability).

### 3.12. Cellular Antioxidant Assay (CAA) in an Intestinal Cell Model

The cellular antioxidant assay was performed following the methods of Wan et al. [51] and Kellett et al. [49], with modifications. Briefly, the Caco-2 cells were cultivated in growth medium and seeded in a 96-well black plate with a clear bottom at a density of 6.5 × 10^4^ cells/well for 48 h to reach confluence. On the day of the assay, the medium was discarded, and the cells were washed with 100 μL D-PBS. Then, the cells were incubated for 1 h at 37 °C with different concentrations of 3 kDa permeate of legume digestate (3000, 3500 and 4000 µg peptides/mL) diluted in DMEM containing 25 μM (final concentration) of Dichloro-dihydro-fluorescein diacetate (DCFH-DA), which is used as a marker of intracellular oxidation. Once DCFH-DA enters the cells, it is diacetylated by intracellular esterase into 2′,7′-dichlorodihydrofluorescein (DCFH) that is more easily oxidizable. The 3 kDa permeate of blank digestions (digestion solutions and enzyme without legume flour) was used as a control, since the digestion medium was shown to interfere in the assay. The DMEM was used as the blank. The DCFH-DA (25 μM final concentration) was also added to the control and blank wells. After the incubation period, the samples were discarded, and 100 μL of a 600 μM AAPH solution, a peroxyl radical initiator, prepared in Hank’s Balanced Salt Solution (HBSS) was added to the sample and control wells. The HBSS without AAPH was added to the blank wells. Once the DCFH was oxidized into dichlorofluorescein (DCF) by the free radicals, the probe became fluorescent. The fluorescence (λ_ex_ = 485 nm, λ_em_ = 520 nm) was reordered every minute for 1 h at 37 °C, using a Synergy HTX plate reader (Bio-Tek, Winooski, VT, USA). The antioxidant capacity was expressed as the Cellular Antioxidant Activity (*CAA*) unit, which was calculated as follows:

$$\mathrm{CAA}\;\left(\%\right)=\left(1-\frac{\int SA}{\int CA}\right)\times 100$$

where *SA* is the sample curve of relative fluorescence over time and *CA* is the control curve of relative fluorescence over time. The area under the curve of the samples and controls were calculated by the Gen5 software (Bio-Tek, Winooski, VT, USA) after the blank and initial fluorescence reading subtraction.

### 3.13. Dipeptidyl Peptidase-IV (DPP-IV) Inhibition in an Intestinal Cell Model 

A cellular model was also used to verify the inhibitory effect of the faba bean-, pea- and soy-derived peptides against DPP-IV in more physiologically relevant conditions. The experiment was carried out as described by Lammi et al. [84], with modifications. The Caco-2 cells were plated in a growth medium 48 h before the assay at a density of 5·10^4^ cells/well in a 96-well black plate with a clear bottom. On the day of the assay, the medium was discarded, and cells were washed with 100 μL D-PBS. The cells were then incubated with different concentrations of 3 kDa permeate of legume digestate (3000, 3500 and 4000 µg/mL) diluted in DMEM, for 2 h at 37 °C. The DMEM was used as the control. After incubation, the medium was discarded, the cells were washed with D-PBS and 100 μL of 50 μM Gly-Pro-AMC prepared in D-PBS was added per well. The PBS without the Gly-Pro-AMC was used for the background subtraction. The plate was incubated for 10 min at 37 °C and the fluorescence (λ_excitation_ = 350 nm, λ_emission_ = 450 nm) was recorded, using a Synergy HTX plate reader (Bio-Tek, Winooski, VT). The DPP-IV inhibition was expressed as follows, after background subtraction:

$$\mathrm{DPP}-\mathrm{IV}\;\mathrm{Inhibition}\;\left(\%\right)=\left(1-\frac{RFU_{sample}}{RFU_{control}}\right)\times 100$$

where *RFU_sample_* is the fluorescence of the samples and *RFU_control_* is the fluorescence of the control.

### 3.14. Faba Bean Peptides Fractionation and Sequencing by Mass Spectrometry

Based on the results of the in vitro and cell culture bioactivity assays, the 3 kDa permeates of the Fabelle digestates were further fractionated by size exclusion chromatography, as described in Section 3.6.1. The obtained fractions were freeze-dried and tested again for their antioxidant and antihypertensive activity. The peptides present in each fraction were identified by tandem mass spectrometry. To do so, the freeze-dried samples were solubilized in 5% (*v/v*) acetonitrile and 0.2% (*v/v*) formic acid and loaded onto a C18 precolumn (0.3 mm × 5 mm) followed by separation on a reversed-phase column (150 μm × 150 mm) with a linear gradient from 10% to 30% (*v/v*) acetonitrile and 0.2% (*v/v*) formic acid at a 600-nL/min flow rate for 56 min, using an Ultimate 3000 HPLC system (Eksigent, Dublin, CA, USA) connected to a Q-Exactive Plus mass spectrometer (Thermo Fisher Scientific, San Jose, CA, USA). Each of the full MS spectrum that was acquired at a resolution of 70,000 was followed by 12 tandem-MS (MS-MS) spectra on the most abundant multiple-charged precursor ions. The tandem-MS experiments were performed using collision-induced dissociation (HCD) at a collision energy of 27%.

All of the MS/MS data were analyzed using PEAKS Studio (Bioinformatics Solutions, Waterloo, ON, Canada; version 10.6). The PEAKS Studio search settings were: Vicia faba database (UniProt/SwissProt); nonspecific digestion enzyme; fragment ion mass tolerance of 0.0100 Da; parent ion tolerance of 10.0 ppm; carbamidomethylation of cysteine as a fixed post-translational modification and acetylation, oxidation, deamidation and phosphorylation as variable post-translational modifications.

Scaffold (version 5.0.1, Proteome Software Inc., Portland, OR, USA) was used to validate the MS/MS-based peptide and protein identifications. The peptide identifications were accepted if they could be established at a greater than 95.0% probability by the Peptide Prophet algorithm [85] with Scaffold delta-mass correction. The protein identifications were accepted if they could be established at a greater than 99.0% probability and contained at least one identified peptide. The protein probabilities were assigned by the Protein Prophet algorithm [86]. 

The faba bean peptides identified by mass spectrometry were searched in the BIOPEP-UWM database to screen for potential bioactive properties.

### 3.15. Statistical Analysis 

Each analysis was performed in triplicate and the results were expressed as mean ± standard deviation (SD). The data were analyzed through analysis of variance (ANOVA) (*p* < 0.05) followed by the Tukey’s honest significant difference (HSD) post-hoc test (*p* < 0.05), using the XLSTAT software (Addinsoft, NY, USA) add-on to Microsoft Excel (Redmond, WA, USA) to determine the significant differences. The Pearson correlation coefficients were calculated to investigate the relationship between free radical scavenging capacities determined by different assays and the relationship between the antioxidant activities in the 3 kDa permeates of legume digestate and the total phenolic content (TPC).

## 4. Conclusions

In this study, the beneficial health potential of faba bean flour was investigated and compared to two commonly used legumes (soy and pea) through the screening of bioactive properties resulting from the physiological context of the in vitro gastrointestinal digestion. The in vitro assays revealed that the faba bean flour digestates had either a similar or better bioactive activity compared to the pea digestates, and a similar or lower bioactive activity compared to soy. Nonetheless, the faba bean varieties showed a higher antioxidant activity and antidiabetic activity in the cell-based assays, which suggest that the faba bean peptides may have a better bioavailability or a better activity in vivo. This hypothesis will require further confirmation. Fabelle, one of the three studied faba bean varieties, stood out for its higher antioxidant and antihypertensive activity. Eleven peptides with excellent in silico predicted activity were identified by mass spectrometry, which confirm that the peptide played an important role in the observed bioactive activities. As there is a growing interest for health-promoting functional food ingredients in the food industry [87], our results demonstrate that faba beans have an excellent bioactive potential that complements their nutritional interest quality and therefore present a high potential for use in the development of new functional and nutraceutical ingredients in food applications. Further investigation of these health-promoting bioactivities with in vivo models and in humans are required to confirm these findings.

## Figures and Tables

**Table 1:** Statistical significance of the studied digestion conditions and faba bean peptide concentration on cell viability as assessed by two-way ANOVA.

Factor	*p*-Value ^1^
***Bile salt***	
Bile salt concentration	0.285
Peptide concentration	**<0.0001**
Bile salt concentration × Peptide concentration	0.774
***Intestinal enzyme***	
Intestinal enzyme used	**<0.0001**
Peptide concentration	**<0.0001**
Intestinal enzyme used × Peptide concentration	**<0.0001**
***Protease inhibitor***	
Protease inhibitor concentration	**<0.0001**
Peptide concentration	**<0.0001**
Protease inhibitor concentration × Peptide concentration	**0.004**

^1^ Values significant at the 5% level are printed in bold-faced type.

**Table 2:** Composition analysis of the 3 kDa permeate of legume digestates.

	% Proteins	Total Polyphenol (mg. Gallic Acid Equivalent/g)	Total Carbohydrates (g Glucose Equivalent/100 g)
	(Dry Base)		
Fabelle	34.37 ± 0.22 ^b^	4.81 ± 0.19 ^a^	43.5 ± 1.6 ^b^
Malik	36.11 ± 0.47 ^c^	4.97 ± 0.25 ^a^	44.0 ± 2.0 ^b^
Snowbird	38.42 ± 0.27 ^d^	4.80 ± 0.31 ^a^	45.2 ± 0.5 ^b^
Amarillo	32.87 ± 0.17 ^a^	4.43 ± 0.17 ^a^	46.7 ± 1.5 ^b^
AAC-26-15	54.49 ± 0.54 ^e^	4.81 ± 0.51 ^a^	16.6 ± 0.8 ^a^

Data are expressed as mean ± standard deviation and means in a column without a common letter differ (*p* <0.05) as analyzed by one-way ANOVA and the Tukey’s test.

**Table 3:** Antioxidant activity of faba bean (variety Fabelle) peptide-enriched fractions (F1, F2 and F3).

	ABTS ^1^ (% Scavenging)	Iron Chelating ^2^ (% Chelating)	ORAC (µmol Trolox eq/mg Peptides)
F1	41.5 ± 10.4 ^c^	67.6 ± 2.7 ^b^	0.7 ± 0.5 ^b^
F2	98.9 ± 0.8 ^a^	54.1 ± 1.8 ^c^	3.2 ± 0.6 ^a^
F3	71.4 ± 15.3 ^b^	88.5 ± 1.9 ^a^	3.1 ± 0.1 ^a^

^1^ Fractions were tested at 40 µg/mL; ^2^ Fractions were tested at 5 µg/mL; Data are expressed as mean ± standard deviation and means with different letter in a column are statistically different (*p* <0.05) as analyzed by one-way ANOVA and the Tukey’s test.

**Table 4:** LC-MS/MS identified faba bean peptides and their respective precursor proteins found in the peptide-enriched fractions of the 3 kDa permeate of Fabelle in vitro gastrointestinal digestate.

Peptide Sequence	Fraction	Observed Mass (Da)	Calculated Mass (Da)	ppm ^1^	Precursor Protein	Protein Accession Number	Fragment Location
N^2^YDEGSEPR	F1	1066.421	1066.420	0.43	Convicilin	B0BCL8	29–37
PVNRPGEPQ	F1	992.507	992.504	2.60	Vicilin	I0B569	152–160
LDNIN^2^ALEPDH	F1	1250.578	1250.578	−0.19	Legumin B	P05190.1	35–45
TETWNPNHPE	F1	1223.522	1223.521	1.21	Legumin B	P05190.1	52–61
TETWNPNHPEL	F1	1336.606	1336.605	0.88	Legumin B	P05190.1	52–62
EEEDEDEPR	F1	1146.436	1146.431	3.64	Legumin	Q43673	327–335
KEEEDEDEPR	F1	1274.530	1274.526	3.06	Legumin	Q43673	326–335
VIPTEPPH	F1	888.470	888.471	−0.70	Tonoplast intrinsic protein 32	A0A024NRI7	155–162
VIPTEPPHA	F1	959.508	959.508	−0.13	Tonoplast intrinsic protein 32	A0A024NRI7	155–163
VVIPTEPPHA	F1	1058.577	1058.576	0.55	Tonoplast intrinsic protein 32	A0A024NRI7	154–163
VVIPTEPPH	F1 and F2	987.540	987.539	1.07	Tonoplast intrinsic protein 32	A0A024NRI7	154–162

Amino acids are abbreviated with 1 letter code; ^1^ Mass error was expressed in ppm and calculated as follow: $\frac{Oberserved\;mass-Calculated\;mass}{Obserevd\;mass}\times 10^{6}$; ^2^ Deamidation of asparagine residue.

**Table 5:** In silico prediction of bioactive properties of the LC-MS/MS identified faba bean peptides using the BIOPEP-UWM database.

Peptide Sequence	Fraction	Potential Bioactivity ^1^	Bioactive Fragments	A ^2^	B ^3^ (μM^−1^)
NYDEGSEPR	F1	ACE inhibitor	PR, GS, EG, NY	0.44	0.03
		Stimulating ^5^	SE	0.11	.
		DPP-IV inhibitor	EP, EG, NY, YD	0.44	.
		DPP-III inhibitor	PR	0.11	.
PVNRPGEPQ	F1	Anti-amnestic	PG	0.11	.
		ACE inhibitor	GEP, RP, GE, PG, PQ	0.56	0.36
		Antithrombotic	PG	0.11	.
		Regulating ^4^	PG	0.11	.
		DPP-IV inhibitor	RP, EP, GE, NR, PG, PQ, PV, VN	0.89	4.96
		DPP-III inhibitor	GE	0.11	.
		Renin inhibitor	NR	0.11	.
LDNINALEPDH	F1	ACE inhibitor	ALEP	0.09	1.44 × 10^−5^
		DPP-IV inhibitor	EP, AL, DN, IN, NA	0.45	1.03 × 10^−4^
TETWNPNHPE	F1	ACE inhibitor	TE, HP	0.2	.
		Antioxidant	TW	0.1	.
		Alpha-glucosidase inhibitor	PE	0.1	3.99 × 10^−6^
		DPP-IV inhibitor	HP, NP, WN, ET, NH, PN, TE, TW	0.8	3.35 × 10^−3^
		DPP-III inhibitor	HP, PE	0.2	.
TETWNPNHPEL	F1	ACE inhibitor	TE, HP	0.18	.
		Antioxidant	EL, PEL, TW, TETWNPNHPEL	0.36	.
		Alpha-glucosidase inhibitor	PE	0.09	3.63 × 10^−6^
		DPP-IV inhibitor	HP, NP, WN, ET, NH, PN, TE, TW	0.73	3.04 × 10^−3^
		DPP-III inhibitor	HP,PE	0.18	.
EEEDEDEPR	F1	ACE inhibitor	PR	0.11	0.03
		Stimulating ^5^	EEE, EE	0.33	.
		DPP-IV inhibitor	EP	0.11	.
		DPP-III inhibitor	PR	0.11	.
KEEEDEDEPR	F1	ACE inhibitor	PR, KE	0.2	0.02
		Stimulating ^5^	EEE, EE	0.3	.
		DPP-IV inhibitor	EP, KE	0.2	.
		DPP-III inhibitor	PR	0.1	.
VIPTEPPH	F1	ACE inhibitor	IP, TE, PT, PP, PH	0.63	9.62 × 10^−4^
		Alpha-glucosidase inhibitor	PP	0.13	6.93 × 10^−6^
		DPP-IV inhibitor	PP, IP, EP, PH, PT, TE, VI	0.88	3.26 × 10^−4^
VIPTEPPHA	F1	ACE inhibitor	IP, TE, PT, PP, PH	0.56	8.55 × 10^−4^
		Antioxidant	PHA	0.11	.
		Alpha-glucosidase inhibitor	PP	0.11	6.16 × 10^−6^
		DPP-IV inhibitor	PP, HA, IP, EP, PH, PT, TE, VI	0.89	2.90 × 10^−4^
VVIPTEPPHA	F1	ACE inhibitor	IP, TE, PT, PP, PH	0.5	7.69 × 10^−4^
		Antioxidant	PHA	0.1	.
		Alpha-glucosidase inhibitor	PP	0.1	5.55 × 10^−6^
		DPP-IV inhibitor	PP, VV, HA, IP, EP, PH, PT, TE, VI	0.9	2.61 × 10^−4^
VVIPTEPPH	F1 and F2	ACE inhibitor	IP, TE, PT, PP, PH	0.56	8.55 × 10^−4^
		Alpha-glucosidase inhibitor	PP	0.11	6.16 × 10^−6^
		DPP-IV inhibitor	PP, VV, IP, EP, PH, PT, TE, VI	0.89	2.90 × 10^−4^

Amino acids are abbreviated with 1 letter code; ^1^ Potential bioactivities for each peptide were determined using the BIOPEP-UWM database [69]; ^2^ The parameter A represents the occurrence frequency of a fragment with a given bioactivity: $A=\frac{a}{N}$, where a is the number of fragments with a given bioactivity and N is the number of amino acid residues in the peptide sequence [70]; ^3^ The parameter B represents the potential biological activity of the peptide: $B=\frac{\sum_{i=1}^{k}\frac{a_{i}}{EC_{50}i}}{N}$, where a is the number of repetitions of a given fragment with a given activity, EC_50_ is its respective half maximum activity (μM) and k is the number of different fragments with a given bioactivity and N is the number of amino acid residues [70]. The B parameter is only calculated if EC_50_ data are available. The higher the B value is, the higher the predicted bioactivity is. A and B were automatically computed by the BIOPEP algorithm; ^4^ Peptide regulating the stomach mucosal membrane activity; ^5^ Peptide stimulating vasoactive substance release.

**Table 6:** UniProtKB Protein accession numbers of faba bean, pea and soy main storage proteins used for the in silico analysis.

Faba Bean (*Vicia faba*)			
Proteins		Gene	Accession Number
Legumin (11S)	Legumin B	LEB4	P05190
		LEB2	P16078
		LEB6	P16079
		LEB7	P16080
	Legumin A	A1	Q03971
		A2	Q99304
Vicilin (7S)	Vicilin	.	P08438
	Convicilin	.	B0BCL8
		.	B0BCL7
Pea (*Pisum sativum*)			
Proteins		Gene	Accession Number
Legumin (11S)	Legumin B	LEGJ	P05692
		LEGK	P05693
		LEGB	P14594
	Legumin A	A	P02857
		A2	P15838
Vicilin (7S)	Vicilin	.	P13918
	Convicilin	CVA	P13915
		CVB	P13919
Soy (*Glycine max*)			
Proteins		Gene	Accession Number
Glycinin (11S)	Glycinin-G1	GY1	P04776
	Glycinin-G2	GY2	P04405
	Glycinin-G3	GY3	P11828
	Glycinin-G4	GY4	P02858
	Glycinin-G5	GY5	P04347
β-Conglycinin (7S)	β-Conglycinin-α’	CG-1	P11827
	β-Conglycinin-α	CG-3	P0DO16
	β-Conglycinin β	CG-4	P25974

## Data Availability

Not applicable.
